# Principles of Genomic Newborn Screening Programs: A Systematic Review

**DOI:** 10.1001/jamanetworkopen.2021.14336

**Published:** 2021-07-20

**Authors:** Lilian Downie, Jane Halliday, Sharon Lewis, David J. Amor

**Affiliations:** 1 Murdoch Children’s Research Institute, Melbourne, Victoria, Australia; 2 Department of Paediatrics, University of Melbourne, Melbourne, Victoria, Australia; 3 Royal Children’s Hospital, Melbourne, Victoria, Australia; 4 Victorian Clinical Genetics Services, Melbourne, Victoria, Australia

## Abstract

**Importance:**

Genomic newborn screening (gNBS) may optimize the health and well-being of children and families. Screening programs are required to be evidence based, acceptable, and beneficial.

**Objectives:**

To identify what has been discovered following the reporting of the first gNBS pilot projects and to provide a summary of key points for the design of gNBS.

**Evidence Review:**

A systematic literature review was performed on April 14, 2021, identifying 36 articles that addressed the following questions: (1) what is the interest in and what would be the uptake of gNBS? (2) what diseases and genes should be included? (3) what is the validity and utility of gNBS? and (4) what are the ethical, legal, and social implications? Articles were only included if they generated new evidence; all opinion pieces were excluded.

**Findings:**

In the 36 articles included, there was high concordance, except for gene disease inclusion, which was highly variable. Key findings were the need for equitable access, appropriate educational materials, and informed and flexible consent. The process for selecting genes for testing should be transparent and reflect that parents value the certainty of prediction over actionability. Data should be analyzed in a way that minimizes uncertainty and incidental findings. The expansion of traditional newborn screening (tNBS) to identify more life-threatening and treatable diseases needs to be balanced against the complexity of consenting parents of newborns for genomic testing as well as the risk that overall uptake of tNBS may decline. The literature reflected that the right of a child to self-determination should be valued more than the possibility of the whole family benefiting from a newborn genomic test.

**Conclusions and Relevance:**

The findings of this systematic review suggest that implementing gNBS will require a nuanced approach. There are gaps in our knowledge, such as the views of diverse populations, the capabilities of health systems, and health economic implications. It will be essential to rigorously evaluate outcomes and ensure programs can evolve to maximize benefit.

## Key Points

**Question:**

Based on current evidence, how should a genomic newborn screening (gNBS) program be designed and implemented?

**Findings:**

This systematic review identified 36 relevant articles to inform important points to consider in the design of a gNBS program. These covered parental interest and uptake of testing; gene selection; clinical validity and utility; and ethical, legal, and social implications.

**Meaning:**

The findings suggest that gNBS should be introduced with key considerations regarding choice, flexible consent, and transparent gene and disease selection, maximizing validity and utility while minimizing uncertainty and reflecting the ethical values of society.

## Teaser

This systematic review identifies what has been discovered following the reporting of the first genomic newborn screening pilot projects and summarizes key points for the design of these programs.

## Introduction

Genomics has altered the landscape of human genetics, with relevance to all medical specialties across all life stages. The technology is being used to improve rates of diagnosis, understand disease prognosis, and develop new therapies.^1,2^ While the concept of precision medicine has evolved to be a realistic goal for individual health care, questions remain about the feasibility and ethics of using genomics for public health benefit.^3^

Genomics in the newborn period as a screening test for asymptomatic babies has the potential to identify hundreds of diseases which, although individually rare, together have significant health and economic burdens on the population.^4^ In addition, newborn genomic samples have the potential to be reanalyzed throughout life for ongoing screening or based on clinical need. Traditional newborn screening (tNBS) has been guided by the principles set out by Wilson and Junger^5^ in the 1960s, when screening programs emerged, targeting severe and treatable pediatric conditions. While the number of conditions screened for has increased, the principles by which conditions are selected has been consistent. Using genomic sequencing for NBS (gNBS) does not necessarily align with these criteria, as it offers the potential to screen for both untreatable and adult-onset diseases.

In response, a reinterpretation of criteria for NBS was published in 2008, taking genetics into account.^6^ Among these criteria, there is no mention of treatment, but the concepts of informed choice and equity of access are introduced. Later, in 2011, the US Centers for Disease Control and Prevention (CDC) published the ACCE tool, a framework through which to evaluate the use of genetic tests in a healthy population, comprising the 4 key domains of analytical validity; clinical validity; clinical utility; and ethical, legal, and social implications (ELSI).^7^ In 2018, a systematic review of the NBS literature^8^ was undertaken to refine the criteria to reflect modern perspectives, but it did not address genomics specifically.

Genomics adds extra complexity and risk to NBS. Most frequently cited are the need for education and consent, risks of loss of autonomy of the child, genetic discrimination, decreased uptake of tNBS programs, the burden of variants of uncertain significance (VUS) and of diseases with decreased penetrance, cost and storage, and privacy of data.^9,10^ But benefits are also cited, such as the ability to screen for more diseases, provide children access to preventative health care measures, and increase the health of the entire family.^11^

Recently, evidence has emerged investigating different aspects of gNBS.^12,13^ Most notably, the first studies offering actual testing of newborns have been completed,^14,15^ providing insights that complement the larger body of hypothetical and laboratory-based studies. Here we summarize the literature generating new evidence on gNBS and, from this, provide practical considerations for designing a gNBS program, reflecting particularly on the existing NBS and ACCE criteria.

## Methods

A literature search of online databases, including PubMed, OVID MEDLINE, OVID Embase, and ProQuest, was performed on April 14, 2021. Searches were performed for all study types published in the English language using MeSH headings and key words: (*Neonatal Screening*) AND (*Genome, Human/ genome/ or exome/*) OR (*sequence analysis, dna/ or dna mutational analysis/*) OR (*genomics/or human genome project/*) OR (*sequence analysis/ or high-throughput nucleotide sequencing/ or molecular sequence annotation/*). Articles were screened by title and abstract for relevance. Full text articles were then assessed for eligibility. Reference lists of all included articles that met criteria were reviewed to identify additional papers for inclusion.

Inclusion criteria were established, which included original studies that addressed 4 key areas of investigation: (1) parental interest and uptake, (2) disease and gene selection, (3) validity and utility, and (4) ELSI. We excluded conference abstracts. Opinion pieces were also excluded on the basis that they did not provide new evidence. A list of identified articles is provided in eTable 1 in the Supplement. Furthermore, articles investigating genomic sequencing as a second-tier test to follow up existing NBS results were excluded on the basis that these represent genomics for a diagnostic purpose.^10^ This incorporated literature regarding NBS for cystic fibrosis. Articles on alternative genetic technology, such as targeted microarray, T-cell receptor excision circles (TREC), and genomewide association studies (GWAS) were also excluded. These technologies are either applied to specific disease groups (eg, TREC for immunodeficiencies) or provide screening regarding a susceptibility (eg, GWAS for likelihood of developing type 1 diabetes). These analyses raise different issues to those encountered when considering genomic sequencing for monogenic disease. Finally, we excluded articles assessing the feasibility of using stored dried blood spot samples for DNA extraction, as we determined that the ability to perform sequencing on DNA extracted from small amounts of blood or saliva has superseded the relevance of this as a method.

Data were extracted by standard form from all studies according to the Preferred Reporting Items for Systematic Reviews and Meta-analyses (PRISMA) reporting guideline.^16^ Recorded information included first author, year of publication, location, study design, sample size, study population, scope and method of testing, outcome measures, key results, and strengths and limitations of the study (eTable 2 in the Supplement).

## Results

**Figure 1.**
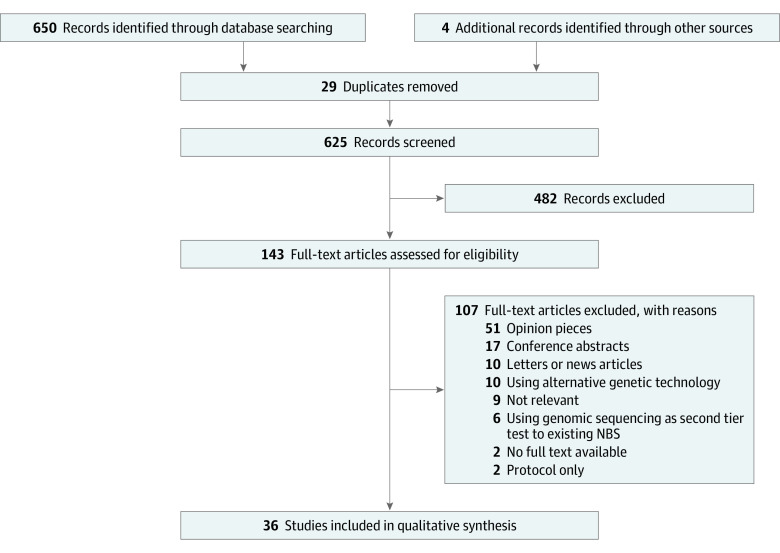
Study Flowchart NBS indicates newborn screening.

A flow diagram outlining the literature review process is presented in Figure 1. The search strategy yielded 650 articles. After records were screened for relevance and duplicates removed, 143 remained for assessment. Overall, 107 records were excluded, leaving 36 studies. These were collated into 4 categories, as detailed in the Table and summarized in what follows.^14,15,17,18,19,20,21,22,23,24,25,26,27,28,29,30,31,32,33,35,36,37,38,39,40,41,42,43,44,45,46,47,48,49,50,51^

**Table.** Summary of Included Studies

Source	Country	Sample size and study population	Key results
**Parental interest and uptake**			
Bombard et al,^17^ 2014	Canada	1213 Adults from the general population	Parents felt less responsibility to have testing using gNBS compared with tNBS. Parents were less likely to participate in screening compared with tNBS (80% vs 94%). Study concluded that offer could reduce uptake of tNBS.
DeLuca,^18^ 2018	United States	88 Families in pediatrician waiting rooms	Overall, 76% knew very little about NBS; 78% wanted face-to-face consent; 97% wanted to screen for as many conditions as possible; and 84% thought screening should be offered for untreatable disorders.
Goldenberg et al,^19^ 2014	United States	1539 Parents	Overall, 74% of parents were somewhat or definitely interested. Most preferred the offer be made by a pediatrician. The most important factors were accuracy of the test and potential for preventing or decreasing a child’s chance of developing disease.
Joseph et al,^20^ 2016	United States	26 Pregnant woman and 5 parents of children with an immune disorder	Participants agreed that parents should be informed and involved in gNBS decisions, potentially prenatally, when they are more likely to be engaged. Mixed views about scope of results. Concern among parents about expansion and consent decreasing uptake of tNBS.
Kerruish,^21^ 2016	New Zealand	15 Parents whose child had screened as high risk for type 1 diabetes in a previous study	Very low level of worry or impact on parenting from previous testing. Concern about gNBS and the timing; consensus about not being in newborn period. Participants suggested that parents have choice around scope of gNBS.
Lewis et al,^22^ 2016	United States	33 Couples pregnant or with a newborn were interviewed, and 1289 parents of children <5 y were included in a DCE	Interview data helped to inform a decision aid and shared parental tool. DCE showed that likelihood of developing disease was most important to parents when choosing diseases to test.
Paquin et al,^23^ 2018	United States	1000 Women pregnant or planning pregnancy	Randomized to education only or education plus values clarification exercise. Those who did the values clarification exercise were more deeply engaged and had stronger intentions to consent to gNBS.
Tarini et al,^24^ 2009	United States	1342 Adults	One-third thought conditions should be screened for only if treatment was available, one-third thought conditions should be screened for even without available treatment, and remainder had no opinion. The Hispanic population was more in favor of testing with no treatment available.
Ulm et al,^25^ 2015	United States	113 Genetic health professionals	Overall, 85% felt gNBS should not be used currently; 76% believed it will be used in this setting in the future; 87% felt parents should be able to choose subsets of results; and 94% felt there needed to be active consent.
Waisbren et al,^26^ 2015	United States	514 Parents within 48 h of birth	Parents reported being not at all (6%), a little (11%), somewhat (37%), very (28%), or extremely (18%) interested in gNBS. Parents were less interested if any health concerns were raised for baby.
Waisbren et al,^27^ 2016	United States	663 Parents completed follow-up surveys from previous study^21^	At a 2-28 mo follow-up, 76% still had some interest; those interested had higher stress ratings on the Parenting Stress Index. There was more interest if any health concerns had been raised for baby.
Etchegary et al,^28^ 2012	Canada	648 Individuals from the general population and prenatal classes	Results from first section of survey (ie, attitudes toward expansion for 3 conditions and reasons): 80% interested in the gNBS, 95% thought it should be offered even if they would decline. Attitudes toward expanded screening were positive, but slightly less positive in parents compared with general population.
Etchegary et al,^29^ 2012	Canada	648 Individuals from the general population and prenatal classes	Results of second section of survey (ie, open questions about inclusion of conditions, risk and benefits): 93% agreed that informed consent was required; accuracy of gNBS was deemed important by 50%; most thought everything should be offered, 38% only if treatment were available, and 24% only if life-threatening condition.
Genetti et al,^30^ 2019	United States	3860 Families of healthy and unwell newborns	Examination of cohort that declined participation in gNBS. Overall, 10% discharged prior to responding to offer, 80% declined at initial approach for involvement in research, and 10% accepted genetic counseling appointment. Of those who attended counseling, 67% (n = 268) enrolled. Study logistics followed by feeling overwhelmed were top reasons for declining participation.
Downie et al,^15^ 2020	Australia	106 Parents of newborns with congenital deafness	Offered gNBS, and 68% wanted additional information (27%, treatable conditions only; 41%, all information possible). Very low decisional regret among all groups. Less decisional conflict and intolerance of uncertainty in those who chose more information. Feeling overwhelmed most common reason for declining additional information.
**Gene and disease selection**			
Berg et al,^31^ 2016	United States	Random sample of 1000 genes	Metric addressed 5 points: (1) severity of disease, (2) likelihood of disease (penetrance), (3) efficacy of intervention, (4) burden of intervention, and (5) knowledge base. Metric was a transparent and effective tool to assesses actionability of a gene disease pair.
Ceyhan-Birsoy et al,^32^ 2017	United States	1514 Genes	954 Genes met reporting criteria after being assessed for validity of gene-disease association, age of onset, penetrance, and mode of inheritance. Reportable genes were those that cause childhood-onset disease with strong evidence and high penetrance, childhood-onset disease with moderate evidence or penetrance but for which there is actionability, pharmacogenomics association, and carrier status.
Milko et al,^33^ 2019	United States	822 Genes	Combined actionability score^34^ with age of onset and intervention to identify 292 genes that met reporting criteria for gNBS and 125 genes for optional disclosure. Reportable genes for gNBS were those that were pediatric onset with high actionability, optional disclosure genes were those that were pediatric onset with low actionability, adult-onset conditions with actionability, and carrier status.
DeCristo et al,^35^ 2021	United States	309 Genes from 4 gNBS gene lists	Evaluated the overlap of the 4 panels and found overall 82 genes thought to be inappropriate for gNBS and 249 genes deemed to be suitable for gNBS were missing.
**Validity and utility**			
Ko et al,^36^ 2018	Korea	20 Infants with metabolic disease and/or abnormal NBS results	Concluded gNBS would complement tNBS by providing earlier and more accurate diagnosis. Limitation was looking at an affected cohort; therefore, the study does not provide information on utility for a whole population or those who screen negative on tNBS.
Lee et al,^37^ 2019	Korea	48 Babies in intensive care units	Overall, 25 genetic variants were identified in 19 infants, with only 1 definitive diagnosis made. Concluded that gNBS complements tNBS by reducing follow-up investigations and clarifying diagnoses earlier and faster.
Narravula et al,^38^ 2017	United States	Genomics results over a 10-y period in 3 disorders from a single laboratory	17 VUS results were reclassified as a result of new information in the literature or in public databases. Many of these could have been classified more accurately with biochemical data. Concluded that avoiding VUS results in gNBS will occur from close liaison with clinical team and biochemical and molecular laboratories.
Pavey et al,^39^ 2017	United States	1349 Newborn-parent trios recruited prenatally	A total of 5 infants were computer-predicted to have immunodeficiency compared with 1 geneticist prediction. Overall, 29 children had features of immunodeficiency, of whom 3 had pathogenic variants. Screening for immunodeficiency would be augmented using gNBS.
Bhattacharjee et al,^40^ 2015	United States	36 Samples from infants known to have a condition detected by tNBS	Genomics accurately identified 27 of 36 conditions (75%) using automated approach and 32 (89%) after manual clinical input was added. Targeted panel had benefit of higher coverage and faster turn-around time.
Bodian et al,^41^ 2016	United States	1696 Neonates with NBS data, correlating genomic data	Overall, 89% (35) true positives and 99% (>45 000) true negatives were correctly called by both technologies. There were 513 results in disagreement (409 due to VUS variant). Concluded the technologies are complementary: no result was uncertain by both methods. A total of 3 cases were missed by genomics.
Ceyhan-Birsoy et al,^14^ 2019	United States	159 Neonates well and unwell, plus 85 parents.	Overall, 10 well and 5 unwell infants had a returnable result, and 3 of 85 parents had cancer predisposition result returned. Difficulty in interpretation of variants in early infancy with no phenotype. Reporting of genes with incomplete penetrance. Detected 3 conditions missed by tNBS.
Solomon et al,^42^ 2012	United States	3 Newborns with normal NBS with clinical diagnosis of VACTERL association	All 3 participants had carrier results identified. No genomics diagnoses made.
Yeh et al,^43^ 2021	United States	Model of gNBS for cancer predisposition syndromes	Concluded that population-based gNBS for cancer predisposition syndromes would reduce pediatric mortality and is likely to be cost-effective.
Wojcik et al,^44^ 2021	United States	159 Neonates in BabySeq project	gNBS results were compared with tNBS results. The technologies were found to be complementary.
**Ethical, legal, and social implications**			
Bunnik et al,^45^ 2013	Netherlands	Expert recommendations	Emphasized importance of informed consent. Child’s right to self-determination means that only childhood-onset disorders should be considered and direct-to-consumer tests should not be available to children. Recommend generic but categorized or differentiated consent for different disease types.
Frankel et al,^46^ 2016	United States	Empirical evidence of psychosocial impact	Domains identified: child vulnerability; parent-child bonding; self and partner blame. Outlined how these will be evaluated in the BabySeq study.
Friedman et al,^47^ 2017	Canada	Global Alliance Pediatric Task Team recommendations	Summary of recommendations: equal access; public data sharing for accurate interpretation of variants; only newborn treatable disease; all appropriate follow-up available; offered in addition to current screening; only replaced if proven increased specificity and sensitivity; and clinical utility and cost-effectiveness must be demonstrated
Golden-Grant et al,^48^ 2015	United States	2 Case reports of population screening identifying adult-onset Pompe disease	Issues identified and discussed: child’s loss of decision-making capacity, potential stress of knowledge, and equity of care and access.
King and Smith,^49^ 2016	United States	Analysis of current laws and application to gNBS	Suggests 3 options for introducing gNBS: use as second tier or report very targeted results and discard the rest; offer parents 1 y to have raw data transferred; or offer opt in analysis.
Holm et al,^50^ 2019	United States	Change in protocol of BabySeq study	Best interests of child vs best interests of family.
Ross and Clayton,^51^ 2019	United States	Discussion of family benefit	Refutes interests of family as a reason to expand newborn screening results.

Abbreviations: DCE, discrete choice experiment; gNBS, genomic newborn screening; tNBS, traditional newborn screening; VACTERL, vertebral anomalies, anal atresia, cardiac defects, tracheoesphegal fistula, renal anomalies, and limb abnormalities; VUS, variant of uncertain significance.

### Parental Interest and Uptake

Fourteen articles^15,17,18,19,20,21,22,23,24,25,26,27,28,29,30^ addressed parental and population interest in gNBS. Twelve studies^17,18,19,20,21,22,23,24,26,27,28,29^ used a hypothetical offer of gNBS, of which 9 studies^17,18,19,23,24,26,27,28,29^ used a survey or questionnaire methodology and 3 studies^20,21,22^ used interviews. Two studies^15,30^ offered genomic sequencing for newborns and addressed parental uptake and motivations for accepting or declining testing. The populations sampled included parents generally, parents of newborns, pregnant couples, the general adult population, and health professionals. In every study, most respondents were White women.

### Gene and Disease Inclusion

Three studies^31,32,33^ addressed detailed processes for selecting genes and/or conditions to be tested in gNBS. These articles came from 2 US studies; NC NEXUS and Babyseq. They took different approaches to curating genes and arrived at different lists for gNBS: BabySeq with 954 genes compared with 292 genes for NC NEXUS, not all of which were assessed by the BabySeq group and vice versa. A single study^35^ assessed direct-to-consumer gNBS gene lists.

### Validity and Utility

Ten studies^14,36,37,38,39,40,41,42,43,44^ addressed the validity and utility of using genomic sequencing to complement or replace tNBS. Four studies^36,37,43,44^ investigated the use of genomic testing with targeted analysis to complement tNBS. Only 3 studies^14,41,44^ tested a cohort of well infants, while the remainder selected cohorts with findings on tNBS. Two studies^39,41^ used first-pass automation to identify pathogenic and likely pathogenic variants. One study^43^ was a simulated model of gNBS for cancer predisposition syndromes.

### ELSI

Seven articles^45,46,47,48,49,50,51^ were identified that evaluated the ELSI. Two studies^45,52^ provided expert recommendations, guidelines, and considerations on the ethical aspects of gNBS broadly, generated by consensus from groups of individuals with expertise in the relevant fields of genomics and ethics. One article^46^ reviewed the literature to determine how to assess the psychosocial outcomes of their study in gNBS (BabySeq). Three articles^48,51,53^ used case studies to highlight and identify important ethical considerations, particularly around the scope of results returned. Two of these^51,53^ were about the same case from the Babyseq study and present different arguments around the ethical approach to gNBS. A single study^49^ examined the US legal framework, regulations, and constitutional underpinnings of existing NBS programs and how gNBS might fit within or challenge these.

## Discussion

**Figure 2.**
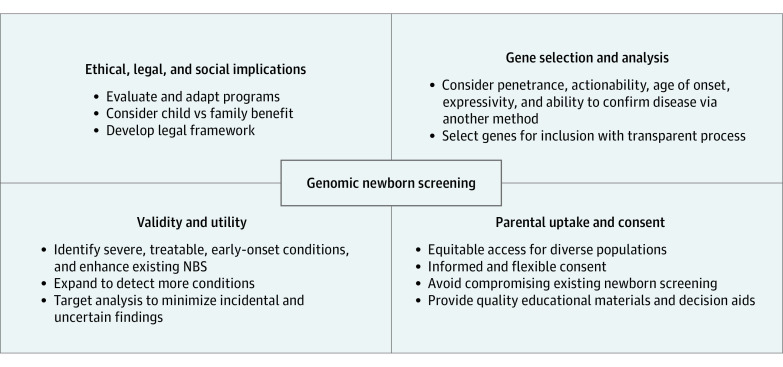
Factors Identified for Consideration in Designing a Genomic Newborn Screening (NBS) Program

Based on the 4 identified areas, we developed a summary of the considerations for a gNBS program. These are illustrated in Figure 2.

### Parental Interest and gNBS Uptake

There was a high level of interest (>60%) in response to both hypothetical and actual offers of gNBS; however, there was considerable variation in the desired scope of results to be returned. While parental support for expanding NBS to look at untreatable disorders was found,^18,24^ there was less support for identifying genetic risk factors for disease.^54^ Consistently, the highest importance was placed on whether the test is accurate at predicting disease.^54,55^ In addition, while parents overwhelmingly wanted active participation in consent,^28,29^ there was evidence that the introduction of gNBS and the need for more detailed consent would decrease participation in tNBS.^17,20,21^ This argues against the integration of genomic testing into existing NBS programs because it may cause harm through lower uptake, leading to missed diagnoses of treatable conditions. Furthermore, in support of separating gNBS from tNBS, 1 study^15^ found that real-time uptake of additional genomic information was lowest in parents with infants younger than 3 months. However, it is notable that in the study that offered gNBS within 48 hours of birth, no parent declined tNBS, indicating that concerns about gNBS affecting the uptake of tNBS programs may be unfounded.^26^ In another cohort, followed up over a 2-year period, the level of interest in gNBS remained high, with interest increasing over time among parents who had health concerns for their child.^26,27^

In support of offering gNBS separate from tNBS is the recognized need for education and informed consent, a point raised in multiple qualitative studies.^20^ Despite education provisions, parents may not fully grasp the possibility of uncertainty that can be raised by genomic testing. Several studies have identified the importance of using decision aids to increase education and understanding of genomic testing.^23,55^ Educational resources should ideally be interactive and adaptable to allow for flexible consent. Historically, face-to-face genetic counselling would have been considered the optimum approach; however, with the increasing use of information technology it is more likely that these resources will become the frontline of information provision in population-based screening programs.

A single study^25^ investigated the opinions of health care professionals working in the field. It found that health care professionals largely agreed with the views of parents, ie, that informed consent is necessary and that gNBS should not be compulsory or opt out as tNBS is in many countries.

Actual uptake was similar between the 2 studies that offered gNBS to parents, ie, 67% (268 of 402)^30^ and 68% (72 of 106),^15^ despite these being different cohorts, with 1 comprising a combination of well babies and very unwell babies admitted to neonatal intensive care unit^30^ and the other comprising systemically well children with congenital deafness.^15^ A similarity between these studies is that both had already consented participants to research, and therefore, these results may not be generalizable.

In summary, public views about gNBS mostly support its introduction and the premise of expanding what is looked for in tNBS. The collated data suggest a move away from the requirement that a treatment be available for all conditions. There is also a recognition of the importance of personal utility to families and individuals, which is increasingly factored into health care decisions and does not always align with clinical utility.^34,56^

### Selection of Genes and Conditions

The scope of gNBS is an important consideration in its evaluation. There is no consensus in the literature about which diseases and genes should be included. Evidence suggests that parents value test accuracy and knowledge about the condition,^54,55^ indicating that disease association and penetrance could be considered a priority above actionability. Increasingly, open-source shared platforms may be used to provide consistency as to gene list analysis.^57^

The NC NEXUS group^31,33^ used a metric that generated a score for each gene-disease pair, representing medical actionability. They combined this score with the age of onset of that condition to bin gene-disease pairs. The first bin included conditions that have childhood onset and are medically actionable and were therefore deemed appropriate for gNBS. They then used this metric to assess the suitability of direct-to-consumer gNBS.^35^ In contrast, the BabySeq group^32^ started by scoring evidence around gene-disease association and then assessed genes by age of onset and available penetrance data. This resulted in 3 categories for analysis: “Category A: genes included in the Newborn Genomic Sequencing Report (NGSR) with definitive or strong evidence to cause a highly penetrant childhood-onset disorder. Category B: genes included in the NGSR based on actionability during childhood. Category C: genes that did not meet criteria to be returned in the NGSR.”^32^ Both groups agreed that a transparent process for selecting and reporting genes was essential.

Many of the other studies^15,39,40,41^ included details on how they created gene lists for analysis in the methods. These were highly variable according to the criteria selected by the research group.

### Clinical Validity and Utility

Seven studies addressed clinical validity of gNBS vs tNBS by investigating cohorts that had findings on tNBS. These found that the addition of gNBS led to an earlier and faster diagnosis but that it was not superior in terms of sensitivity and specificity. gNBS has been shown to be particularly useful in unwell newborns, whereas tNBS results can be difficult to interpret.^36,37^ Only 3 studies^14,41,44^ tested cohorts of well infants and again supported the idea that tNBS and gNBS are complementary, with overlapping sensitivity and specificity. Other studies^38,42^ addressed whether gNBS could replace tNBS and concluded that the burden of VUS results as well as the difficulty in interpreting them without biochemical data make replacement unfeasible. Proposals to address this included increased integration between clinician and laboratory to make variant interpretation more accurate and therefore lead to fewer VUS results^38^ or pathogenic variant detection only,^42^ which also improves analytical validity.

Regarding clinical utility, there is some consensus that analysis of a targeted gene panel is preferable to broad genomic sequencing, reducing the potential harms from detection of VUS and incidental findings.^39,40^ Two studies^39,41^ used first-pass automation to identify pathogenic and likely pathogenic variants and concluded that this improved efficacy and cost-effectiveness.

Several articles^39,40,42,43^ found that gNBS would significantly increase the number of babies diagnosed due to the ability to detect disorders without a biochemical test, with delayed onset or mild phenotype that are likely to be missed with current screening. For diseases such as immunodeficiencies and cancer predisposition syndromes, there was a postulated cost saving to the health care system in the long term.

Utility—clinical or personal—can be difficult to measure, particularly at a single time, as demonstrated in 1 study^14^ in which several infants were identified as having a pathogenic or likely pathogenic variant in a cardiomyopathy gene with reduced penetrance. Without long-term follow up of large cohorts, it is difficult to predict the positive and negative impacts of this type of result on individuals, families, and the medical system.

Conclusions drawn from the literature are that genomic testing does not currently surpass tNBS in terms of sensitivity, specificity, or cost-effectiveness and, therefore, should not be considered as a replacement. Rather it should be considered complementary or as an independent mode of accessing precision health care for individuals. An important consideration is separating the offers of gNBS and tNBS to avoid jeopardizing the success and efficacy of tNBS. This must be weighed against the benefit of increasing the range of conditions that can be detected in the newborn period, including those that fulfill traditional criteria of being treatable early in life, such as immunodeficiencies. The provision of adequate information and resources for parents to opt in if they deem it beneficial for their own circumstances and beliefs will be an important component of any program.

### ELSI of gNBS

There is consensus in the literature regarding certain ELSI. One is the importance of minimizing psychological harm and stress to parents. A potential approach is to use targeted analysis,^45,46,52^ thereby reducing VUS results and the identification of variants in genes with reduced penetrance. Another is to incorporate detailed informed consent so that parents can understand potential risks and only consent if the positives outweigh the potential harms for them.^45,47,48^ However, it has also been highlighted that more choice is not necessarily better and that more complex choices can undermine comprehension, thereby compromising informed decision making.^45^

A single article^49^ assessed the current US legal framework under which tNBS operates and applied it to gNBS. It concluded that genomic testing should only be used to confirm tNBS results in a targeted way and that further disclosure of genomic newborn data to parents would require specific consent procedures and/or legal refinements.

Three studies^48,50,51^ used individual cases to highlight the ethical and moral issues raised by gNBS, helping to contextualize the considerations. One contentious issue is shifting the goals of NBS from being solely about the child to benefit for the whole family. This has been addressed widely, with most stakeholders placing highest value on the ethical principal of the right of self-determination of the child above the considerations of the family.^45,47,51^ A major issue is the absence of empirical evidence regarding harm or benefit,^46^ with most ELSI literature being based on expert opinion. This is a key area of evaluation for the implementation of gNBS.

### Limitations

Limitations of these findings are that the literature reflects a White, female, and higher socioeconomic status view and that more diversity is required in the data. Furthermore, health care systems across the world have different NBS capabilities,^58^ decreasing the generalizability of these results. In addition, research looking at the burden on the workforce, health economics, and data storage is lacking. The conclusions drawn from this review are subject to evolving research, which is active in this area.

## Conclusions

The literature provided insights into the considerations and design of a gNBS program, highlighting that a nuanced approach is required. With advancements in technology and flexibility in the approach, a gNBS program is achievable and potentially of considerable benefit to the population. This review provided evidence and a practical summary on which to base program design. Regardless of how a gNBS program is offered, it will be essential to rigorously evaluate the outcomes and processes and ensure it is able to evolve to meet the needs of the population it serves.^59^

## Notes

Supplement. **eTable 1.** Reference List of 51 Opinion Pieces Identified in Literature Review **eTable 2. **Data Extraction From Included Studies
