# Supplementary material for: Principles of Genomic Newborn Screening Programs: A Systematic Review
Source: JAMA Netw Open. 2021 Jul 20;4(7):e2114336. doi: 10.1001/jamanetworkopen.2021.14336 (PMC8293022; doi:10.1001/jamanetworkopen.2021.14336)
Supplement: Supplement. — eTable 1. Reference List of 51 Opinion Pieces Identified in Literature Review eTable 2. Data Extraction From Included Studies [file jamanetwopen-e2114336-s001.pdf]

Supplemental Online Content

Downie L, Halliday J, Lewis S, Amor DJ. Principles of genomic newborn screening programs. *JAMA Netw Open.* 2021;4(7):e2114336.
doi:10.1001/jamanetworkopen.2021.14336

eTable 1. Reference List of 51 Opinion Pieces Identified in Literature Review

eTable 2. Data Extraction From Included Studies

This supplemental material has been provided by the authors to give readers additional information about their work.

© 2021 Downie L et al. *JAMA Network Open.*

eTable 1. Reference List of 51 Opinion Pieces Identified in Literature Review

Almannai M, Marom R, Sutton VR. Newborn screening: a review of history, recent advancements, and future perspectives in the era of next generation sequencing. Curr Opin Pediatr. 2016;28(6):694-699.
Almond B. Genetic profiling of newborns: ethical and social issues. Nature Reviews Genetics. 2006;7(1):67-71.
Anonymous. Newborn screening grows up. Nat Med. 2005;11(10):1013.
Anonymous. Sequenced from the start. Nature. 2013;501(7466):135.
Anonymous. Genomic Screening in Newborns Holds Promise, Challenges: Results of the BabySeq Project demonstrate the potential of newborn genomic screening, but challenges remain. American Journal of Medical Genetics Part A. 2019;179(4):519-520.
Bales AM. A summer in research on newborn screening. Wisconsin Medical Journal. 2010;109(4):225-226.
Beckmann JS. Can we afford to sequence every newborn baby's genome? Human mutation. 2015;36(3):283-286.
Berg JS, Agrawal PB, Bailey DB, Jr., et al. Newborn Sequencing in Genomic Medicine and Public Health. Pediatrics. 2017;139(2).
Berg JS, Khoury MJ, Evans JP. Deploying whole genome sequencing in clinical practice and public health: meeting the challenge one bin at a time. Genetics in medicine : official journal of the American College of Medical Genetics. 2011;13(6):499-504.
Berg JS, Powell CM. Potential Uses and Inherent Challenges of Using Genome-Scale Sequencing to Augment Current Newborn Screening. Cold Spring Harbor Perspectives in Medicine. 2015;5(12):05.
Borry P, Senecal K, Knoppers BM. Do it yourself newborn screening. JAMA pediatrics. 2016;170(6):523-524.
Botkin JR, Rothwell E. Whole Genome Sequencing and Newborn Screening. Current genetic medicine reports. 2016;4(1):1-6.
Cabello JF, Novoa F, Huff HV, Colombo M. Expanded Newborn Screening and Genomic Sequencing in Latin America and the Resulting Social Justice and Ethical Considerations. *Int J Neonatal Screen*. Jan 21 2021;7(1)doi:10.3390/ijns7010006
Caggana M, Jones EA, Shahied SI, Tanksley S, Hermerath CA, Lubin IM. Newborn screening: from Guthrie to whole genome sequencing. Public Health Rep. 2013;128 Suppl 2:14-19.
Camelo JS, Jr. From Expanded Neonatal Screening to the Post-Genomic Era. Rev. 2017;35(3):240-241.
Chakravorty S, Hegde M. Inferring the effect of genomic variation in the new era of genomics. Human mutation. 2018;39(6):756-773.
Dickerson JA, Conta JH. Are we ready for newborn genome screening? Clinical Chemistry. 2017;63(3):794.
Dimmock DP, Bick DP. Ethical issues in DNA sequencing in the neonate. Clin Perinatol. 2014;41(4):993-1000.
Evans JP, Berg JS, Olshan AF, Magnuson T, Rimer BK. We screen newborns, don't we?: realizing the promise of public health genomics. Genetics in Medicine. 2013;15(5):332-334.
Feero WG, Guttmacher AE. Genomics, personalized medicine, and pediatrics. Academic pediatrics. 2014;14(1):14-22.
Ficicioglu C. New tools and approaches to newborn screening: ready to open Pandora's box? Cold Spring Harbor molecular case studies. 2017;3(3):a001842.
Francescatto L, Katsanis N. Newborn screening and the era of medical genomics. Seminars in Perinatology. 2015;39(8):617-622.
Friedman E. Next generation sequencing for newborn screening: are we there yet? Genetical research. 2015;97:e17.
Goldenberg AJ, Sharp RR. The ethical hazards and programmatic challenges of genomic newborn screening. Jama. 2012;307(5):461-462.
Holmes D. Europe plays catch-up on neonatal screening as US skips ahead. Nat Med. 2012;18(11):1596.
Howard HC, Knoppers BM, Cornel MC, Wright Clayton E, Senecal K, Borry P. Whole-genome sequencing in newborn screening? A statement on the continued importance of targeted approaches in newborn screening programmes. European Journal of Human Genetics. 2015;23(12):1593-1600.
Johnston J, Lantos JD, Goldenberg A, et al. Sequencing Newborns: A Call for Nuanced Use of Genomic Technologies. Hastings Cent Rep. 2018;48 Suppl 2:S2-S6.
Khoury MJ, McCabe LL, McCabe ER. Population screening in the age of genomic medicine. New England Journal of Medicine. 2003;348(1):50-58.
King JR, Notarangelo LD, Hammarström L. An appraisal of the Wilson & Jungner criteria in the context of genomic-based newborn screening for inborn errors of immunity. *J Allergy Clin Immunol*. Feb 2021;147(2):428-438. doi:10.1016/j.jaci.2020.12.633
King JR, Hammarstrom L. Newborn Screening for Primary Immunodeficiency Diseases: History, Current and Future Practice. Journal of Clinical Immunology. 2018;38(1):56-66.
Kingsmore SF. Newborn testing and screening by whole-genome sequencing. Genetics in Medicine. 2016;18(3):214-216.

© 2021 Downie L et al. *JAMA Network Open.*

Knoppers BM, Senecal K, Borry P, Avard D. Whole-genome sequencing in newborn screening programs. Science translational medicine. 2014;6(229):229cm222.
Landau YE, Lichter-Konecki U, Levy HL. Genomics in newborn screening. Journal of Pediatrics. 2014;164(1):14-19.
Lantos JD. Introduction to Bioethics Special Supplement V: Ethical Issues in Genomic Testing of Children. Pediatrics. 2016;137 Suppl 1:S1-2.
Levy HL. Newborn screening: the genomic challenge. Molecular genetics & genomic medicine. 2014;2(2):81-84.
Linden Phillips L, Bitner-Glindzicz M, Lench N, et al. The future role of genetic screening to detect newborns at risk of childhood-onset hearing loss. Int J Audiol. 2013;52(2):124-133.
Meade C, Bonhomme NF, Terry SF. Newborn screening: Adapting to advancements in whole-genome sequencing. Genetic Testing and Molecular Biomarkers. 2014;18(9):597-598.
Mollison L, Berg JS. Genetic screening: birthright or earned with age? Expert Rev Mol Diagn. 2017.
Phornphutkul C, Padbury J. Large Scale Next Generation Sequencing and Newborn Screening: Are We Ready? Journal of Pediatrics. 2019;209:9-10.
Pollitt RJ. Different viewpoints: International perspectives on newborn screening. Journal of medical biochemistry. 2014;34(1):18-22.
Rego S. Newborn screening in the genomics era. Journal of law and the biosciences. 2014;1(3):369-377.
Reinstein E. Challenges of using next generation sequencing in newborn screening. Genetical research. 2015;97:e21.
Roberts JS, Dolinoy D, Tarini B. Emerging issues in public health genomics. Annual Review of Genomics & Human Genetics. 2014;15:461-480.
Senecal K, Vears DF, Bertier G, Knoppers BM, Borry P. Genome-based newborn screening: A conceptual analysis of the best interests of the child standard. Personalized Medicine. 2015;12(5):439-441.
Sondheimer N. Newborn screening by sequence and the road ahead. Clinical Chemistry. 2013;59(7):1011-1013.
Tarini BA, Goldenberg AJ. Ethical issues with newborn screening in the genomics era. Annual Review of Genomics & Human Genetics. 2012;13:381-393.
Urv TK, Parisi MA. Newborn screening: Beyond the spot. Advances in Experimental Medicine and Biology. 2017;1031:323-346.
van El CG, Cornel MC, Borry P, et al. Whole-genome sequencing in health care: recommendations of the European Society of Human Genetics. European journal of human genetics : EJHG. 2013;21(6):580-584.
Wade CH, Tarini BA, Wilfond BS. Growing up in the genomic era: implications of whole-genome sequencing for children, families, and pediatric practice. Annu Rev Genomics Hum Genet. 2013;14:535-555.
Wilcken B. Newborn screening: how are we travelling, and where should we be going? J Inherit Metab Dis. 2011;34(3):569-574.
Yang L, Chen J, Shen B. Newborn screening in the era of precision medicine. Advances in Experimental Medicine and Biology. 2017;1005:47-61.

© 2021 Downie L et al. *JAMA Network Open.*

eTable 2. Data Extraction From Included Studies

Parental interest and uptake				
Author, year, location	*Study design*	*Sample size, study population*	*Test offered*	*Key results, strengths and limitations*
Bombard[17], 2014, Canada	Prospective cohort study by questionnaire	1213 adults from the general population	Genomics as a hypothetical test; untargeted and targeted	Less perceived parental responsibility to have testing using genomic technology compared to tNBS.<br>Less likely to participate in screening compared to tNBS* (79.6% vs 94.4%).<br>Concluded that offer could reduce uptake of tNBS.
DeLuca[18], 2018, USA	Prospective cohort study by questionnaire	88 parents/families in paediatrician waiting rooms	Exploring the concept of NBS expansion	76% knew 'very little' about NBS.<br>78% wanted face to face consent.<br>97% wanted to screen for as many conditions as possible.<br>84% thought screening should be offered for untreatable disorders.
Goldenberg[19], 2014, USA	Prospective cohort study by online survey	1539 parents	gNBS (WGS) as hypothetical test. Group randomized to WGS with NBS or WGS separately offered by paediatrician	74% of parents somewhat or definitely interested.<br>70% somewhat or definitely interested in the offer being made by pediatrician.<br>Not statistically significant between groups.<br>Most important factors were accuracy of the test and potential for preventing or decreasing a child's chance of developing disease.<br>The lowest proportion of respondents deemed knowing their child is a higher risk of developing certain diseases than other people as very important.
Joseph[20], 2016, USA	Focus group interviews	26 pregnant woman and 5 parents of children with immunodeficiency	Hypothetical WGS for expanded gNBS	Agreement that parents should be informed and involved in NBS decisions, potentially prenatally when they are more likely to be engaged.<br>Mixed views about use of WGS and scope of results. Concern among parents about expansion and consent resulting in higher rate of decliners for tNBS.
Kerruish[21], 2016, NZ	Individual semi structured interviews	15 parents where children had been screened as high risk for developing T1DM in a previous study	Hypothetical WGS for expansion of NBS and experience of genetic testing for a risk or predisposition	Very low level of worry or impact on parenting from previous testing.<br>Concern about WGS and the timing – consensus about not being in newborn period.<br>Choice of what disorders to test for highlighted as important – not blanket approach.
Lewis[22], 2016, USA	Semi structured interviews and DCE*	33 couples pregnant or with a newborn, half had child who had received genetic testing in last 5 years.<br><br>1289 parents of children <5yo for	Hypothetical WES- NBS +/- carrier status, adult onset treatable conditions and childhood conditions untreatable	Interview data helped inform information provided in decision aid and prompted 'shared' parental tool.<br>DCE showed that likelihood of developing disease was most important to parents when choosing diseases to test.

© 2021 Downie L et al. *JAMA Network Open.*

		discrete choice experiment		
Paquin[23], 2018, US	Randomised controlled trial by online questionnaire	1000 women pregnant or planning pregnancy	Hypothetical use of genomic sequencing in newborns and educational materials required for informed consent	Randomised to education only or education plus values clarification exercise.<br>Values clarification affects the strength of beliefs toward a decision, postulating that people engage more deeply when using this method.<br>Those who did values clarification had stronger intentions to consent to genomic sequencing.
Tarini[24], 2009, USA	Prospective cohort study by internet survey	1342 adults	Hypothetical genetic screening for treatable and untreatable childhood and adult onset conditions	1/3 thought conditions should be screened for only if treatment available<br>1/3 even without treatment<br>1/3 no opinion<br>Hispanic population more in favour of testing with no treatment.<br>27.6% definitely or probably interested in predictive genetic testing with uncertainty (uncertain age of onset and or symptoms).
Ulm[25], 2015, USA	Descriptive cross sectional pilot study by survey	113 genetic health professionals	Hypothetical WGS for NBS	85% felt genomics should NOT be used in NBS currently<br>75.7% believe it will be used in this setting in the future<br>87.3% felt parents should be able to choose subsets of results<br>93.7% felt there needed to be active consent
Waisbren[26], 2015, US	Prospective cohort study by survey	514 parents within 48 hours of birth	Hypothetical genomic sequencing for healthy newborn	Parents reported being not at all (6.4%), a little (10.9%), somewhat (36.6%), very (28.0%), or extremely (18.1%) interested.<br>Less interest if any health concern raised re baby.
Waisbren[27], 2016 US	Prospective cohort study by survey	663 parents completed follow up surveys from previous study[20]	Hypothetical genomic sequencing	2-28 month follow up. 76.1% still had some interest, those interested had higher stress rating on the Parenting Stress Index. More interest if any health concern raised re baby.
Etchegary[28], 2012 Canada	Prospective cohort study by survey	648 individuals from the general population and expecting parents from prenatal classes	Expanded gNBS for hearing loss, vision loss and neurological conditions	Results from first section of survey (attitudes toward expansion for these three conditions and reasons)<br>80% interested in the testing<br>95% thought it should be offered even if they would decline<br>Attitudes toward expanded screening were positive, but slightly less positive in parents compared with general population
Etchegary[29], 2012, Canada	Prospective cohort study by survey	648 individuals from the general population and expecting parents from prenatal classes	Expanded gNBS generally	Results of second section of survey (open questions about inclusion of conditions, risk and benefits)<br>93% agreed that informed consent was required. Accuracy of the test was deemed important by half and not important by other half.<br>Majority thought everything should be offered 38% only if treatment available and 24% only if life threatening condition.
Genetti[30], 2019, USA	Randomized	3860 families approached,	WES with targeted analysis	10% discharged prior to responding to offer<br>80% declined at initial approach

© 2021 Downie L et al. *JAMA Network Open.*

	controlled trial	health newborns and newborns admitted to ICU*, examination of cohort that declined participation		10% accepted genetic counseling appointment<br>67% of those who attended counseling enrolled n = 268. 'Study logistics' followed by 'feeling overwhelmed' were top reasons for declining participation.
Downie[15], 2020, Australia	Prospective cohort study by survey	106 parents of newborns with congenital deafness	WES for diagnosis of aetiology of hearing loss and offer of additional information.<br>A - Diagnostic analysis only;<br>B – A+childhood onset conditions with treatment;<br>C – A+B and +childhood onset conditions without treatment	68% wanted additional information<br>B – 27.4%<br>C – 40.6^%<br>Very low decisional regret amongst all groups<br>Less decisional conflict and intolerance of uncertainty in those who chose more information.<br>'Feeling overwhelmed' most common reason for declining additional information.
Gene/Disease selection				
Berg[31], 2016, USA	Gene list curation	Random sample of 1000 genes	Gene disease actionability score	Metric addresses 5 points; severity of disease, likelihood of disease (penetrance), efficacy of intervention, burden of intervention and knowledge base. Metric is a transparent and effective tool to assesses 'actionability' of a gene disease pair
Ceyhan-Birsoy[32], 2017, USA	Gene list curation	1514 genes	Gene disease suitability for reporting in newborn sequencing	954 genes met reporting criteria after being assessed for: validity of gene-disease association, age of onset, penetrance and mode of inheritance. Reportable genes were those that cause childhood onset disease with strong evidence and high penetrance, childhood onset disease with moderate evidence or penetrance but for which there is actionability, pharmacogenomics association and carrier status.
Milko[33], 2019, USA	Gene list curation	822 genes	Gene disease suitability for reporting in newborn sequencing	Combined actionability score with age of onset and intervention to identify 292 genes that met reporting criteria for gNBS and 125 genes for optional disclosure. Reportable genes for gNBS were those that were paediatric onset with high actionability, optional disclosure genes were those that were paediatric with low actionability, adult onset actionable conditions and carrier status.
DeCristo[35], 2021, USA	Gene list comparison	309 genes on 4 commercially available panels	Gene suitability for inclusion on newborn panels using actionability	Evaluated the overlap of the 4 panels and found overall that 82 genes thought to be inappropriate for gNBS, 249 genes deemed to be suitable for gNBS missing.

© 2021 Downie L et al. *JAMA Network Open.*

			tool developed by NC NEXUS team	
Validity and Utility				
Ko[36], 2018, Korea	Prospective cohort study	20 infants with known diagnosis of metabolic disease or abnormal NBS results	NGS panel for 259 actionable diseases, includes CNV* calling in parallel with traditional NBS	17/20 molecular diagnoses, combined with biochemical results.<br>Concluded gNBS would complement tNBS by providing earlier and more accurate diagnosis. Limitation was looking at an affected cohort, therefore does not provide information on utility for a whole population or those who screen negative on tNBS.
Lee[37], 2019, Korea	Prospective cohort study	48 NICU babies with any indication for admission	Targeted genomic panel of 198 genes in parallel with tNBS	25 variants in 19 infants, only 1 definitive diagnosis made. Concludes that gNBS complements traditional NBS by reducing follow up investigations and clarifying diagnoses earlier and faster.
Narravula[38], 2017, US	Retrospective data analysis	All variants identified by sequence analysis over a 10year period in 3 NBS disorders from a single laboratory	Genomic sequencing – reanalysis of variants	17 VUS results were re-classified as a result of new information in the literature or on public databases.<br>Many of these could have been classified more accurately with biochemical data.<br>Concluded that avoiding VUS results in gNBS will occur from close liaison with clinical team, biochemical and molecular laboratories.
Pavey[39], 2017, USA	Retrospective data analysis	1349 newborn-parent trios recruited prenatally	WGS – targeted analysis of 329 immunodeficiency genes with automated primary analysis	5 infants computer predicted to have immunodeficiency, compared with one geneticist prediction. 29 children had features of immunodeficiency of which 3 had pathogenic variants.<br>GNBS would augment screening for immunodeficiency.
Bhattacharjee[40], 2015,USA	Retrospective 'proof of concept study'	36 samples from infants known to have a condition detected by traditional NBS	Targeted panel vs WES looking for<br>126 conditions detectable by tNBS	27/36 initial accurate calling then 32/36 once clinically correlated<br>Targeted panel had benefit of higher coverage and faster turn-around time.
Bodian[41], 2016 USA	Retrospective cohort study	1696 neonates who had NBS data (includes affected and healthy)	WGS trios (done for other studies) for 163 NBS diseases with automated variant calling compared to tNBS	88.6% (35) true positives and 98.9% (45000+) true negatives correctly called by both technologies.<br>513 results where disagreement (409 due to VUS variant).<br>Concluded the technologies are complementary – no result was 'uncertain' by both methods.<br>3 cases missed by WGS
Ceyan-Birsoy[14], 2019, USA	Randomised controlled trial	159 neonates well and unwell, plus 85 parents	GNBS plus indication based reporting of WES	10 well and 5 NICU infants had a returnable result.<br>3/85 parents had cancer predisposition result returned<br>Difficulty in interpretation of variants in early infancy with no phenotype.<br>Reporting of genes with incomplete penetrance. Detected 3 conditions 'missed' by tNBS.
Solomon[42], 2012, USA	Case series	3 newborns with normal	WES with targeted analysis	All 3 participants had carrier results identified.

© 2021 Downie L et al. *JAMA Network Open.*

		NBS with clinical diagnosis of VACTERL	of 151 genes related to tNBS conditions +omniarray (to detect CNV's)	
Yeh[43], 2021, USA	Simulation model	3.7 million newborns in USA included in model screening for cancer predisposition syndromes	Targeted panel of cancer predisposition syndrome genes.	13.3% of newborns would be identified as at risk of a malignancy and undergo surveillance, predicted to reduce mortality of this group by >50%. Health economic modelling indicated this could be cost-effective as the price of sequencing falls.
Wojcik[44], 2021, USA	Randomised controlled trial	159 neonates from Babyseq study	gNBS results compared with tNBS results	Overlap in sensitivity and specificity of technologies – highlighted they are complementary.
Ethical, legal and social implications				
Bunnik[45], 2013, Netherlands	Ethics discussion and recommendations regarding consent	Not applicable	Genomics in neonatal, prenatal and direct to consumer settings	Emphasized importance of informed consent.<br>Child's right to self-determination means that only childhood onset disorders should be considered and direct to consumer tests should not be available to children.<br>Recommend generic but categorized or differentiated consent for different disease types.
Frankel[46], 2016, USA	Literature review	Looking at empirical evidence of actual psychosocial impact to test the theoretically suggested impacts	Genomic information in newborn period	Domains identified:<br>Child vulnerability<br>Parent-child bonding<br>Self and partner blame.<br>Outlined how these will be evaluated in the Babyseq study.
Friedman[47], 2017, Canada	Consensus expert guidelines	Global Alliance Paediatric Task Team recommendations	Genomic sequencing for newborn population screening	Summary of recommendations<br>- Equal access<br>- Public data sharing for accurate interpretation of variants<br>- Only newborn treatable disease<br>- All appropriate follow up available<br>- In addition to current screening<br>- Only replaced if proven increased specificity and sensitivity<br>- Clinical utility and cost effectiveness must be demonstrated
Golden-Grant[48], 2015, USA	Ethics framework	Case report x 2 of population screening identifying adult onset Pompe disease	Carrier screening and NBS using genomic technology	Proband (child's) loss of decision-making capacity<br>Potential stress of knowledge<br>Equity of care and access
King[49], 2016, US	Legal framework governing state based NBS	Analysis of current laws governing NBS and how these might	Genomic screening in all newborns	Suggests 3 options for introducing gNBS<br>1. Use as second tier or report very targeted results and discard the rest<br>2. As above but offer parents 1yr to have raw data transferred

© 2021 Downie L et al. *JAMA Network Open.*

		apply to genomic NBS		3. Offer opt in analysis
Holm[50], 2019, USA	Case report from Babyseq, returning adult-onset findings.	Change in protocol-ethics decision	GNBS (untargeted analysis)	Ethics discussion: best interests of child vs best interests of family.
Ross[51], 2019, USA	Response to case report Babyseq, returning adult-onset findings.	Discussion of the ethical issues surround 'family benefit'	GNBS(untargeted analysis)	Refutes interests of family as a reason to expand to gNBS.

Abbreviations: t*NBS – traditional newborn screening, WGS – whole genome sequencing, gNBS – genomic newborn screening, WES – whole exome sequencing, DCE – discrete choice experiment, ICU – intensive care unit, CNV – copy number variant, VUS – variant of uncertain significance*

© 2021 Downie L et al. *JAMA Network Open.*
